# Dangers of the chronic stress response in the context of the microbiota-gut-immune-brain axis and mental health: a narrative review

**DOI:** 10.3389/fimmu.2024.1365871

**Published:** 2024-05-02

**Authors:** Alison Warren, Yvonne Nyavor, Aaron Beguelin, Leigh A. Frame

**Affiliations:** ^1^ The Frame-Corr Laboratory, Department of Clinical Research and Leadership, The George Washington University School of Medicine and Health Sciences, Washington, DC, United States; ^2^ Department of Biotechnology, Harrisburg University of Science and Technology, Harrisburg, PA, United States; ^3^ The Department of Biotechnology, Johns Hopkins University, Baltimore, MD, United States

**Keywords:** physiological stress, microbiome, mental health, psychopathology, autoimmunity

## Abstract

More than 20% of American adults live with a mental disorder, many of whom are treatment resistant or continue to experience symptoms. Other approaches are needed to improve mental health care, including prevention. The role of the microbiome has emerged as a central tenet in mental and physical health and their interconnectedness (well-being). Under normal conditions, a healthy microbiome promotes homeostasis within the host by maintaining intestinal and brain barrier integrity, thereby facilitating host well-being. Owing to the multidirectional crosstalk between the microbiome and neuro-endocrine-immune systems, dysbiosis within the microbiome is a main driver of immune-mediated systemic and neural inflammation that can promote disease progression and is detrimental to well-being broadly and mental health in particular. In predisposed individuals, immune dysregulation can shift to autoimmunity, especially in the presence of physical or psychological triggers. The chronic stress response involves the immune system, which is intimately involved with the gut microbiome, particularly in the process of immune education. This interconnection forms the microbiota-gut-immune-brain axis and promotes mental health or disorders. In this brief review, we aim to highlight the relationships between stress, mental health, and the gut microbiome, along with the ways in which dysbiosis and a dysregulated immune system can shift to an autoimmune response with concomitant neuropsychological consequences in the context of the microbiota-gut-immune-brain axis. Finally, we aim to review evidenced-based prevention strategies and potential therapeutic targets.

## 1 Introduction

Over 1 in 5 youth and adults live with mental illness in the United States alone, and 1 in 25 live with a serious mental illness (e.g., schizophrenia, bipolar disorder, major depressive disorder (MDD), which further increases risk for a plethora of physical diseases (1). Pharmacotherapy interventions are rapidly evolving but often carry significant side effects, such as weight gain, metabolic dysfunction, extrapyramidal symptoms, and tardive dyskinesia, a drug-induced movement disorder (2). What’s more, many psychiatric medications as well as polypharmacy possess antimicrobial properties, which alter the gut and neural milieu (3). As such, there is a dire need for effective prevention and treatment strategies, not only for the primary illness, but also for the wide array of common comorbidities.

**Figure 1:**
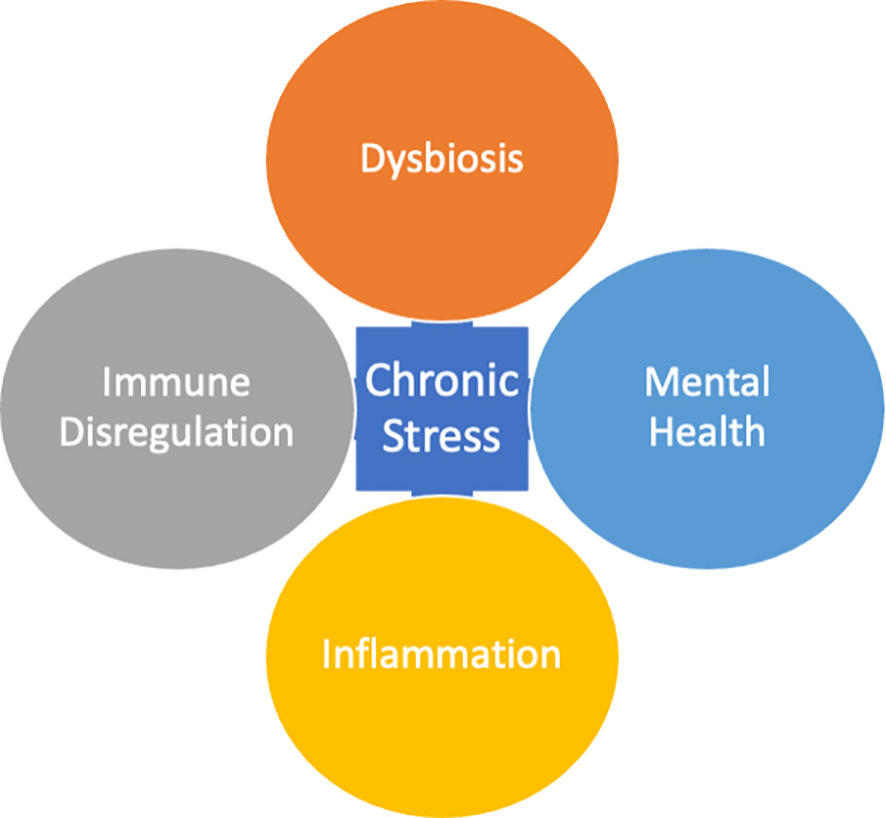
Chronic Stress is a compounded response that occurs in positive feedback loop with Dysbiosis, Immune Dysregulation, Inflammation, and Mental Health.

Since Hans Selye’s (1946) (4) pioneering work on stress and the general adaptation syndrome, stress has become a well-established key risk factor for psychiatric disorders (5). Psychosocial stressors and diathesis stress models are the core of many etiological theories of mental illness (6). More specifically, the pathoetiology of psychiatric disorders continues to unravel complex multidirectional relationships involving neuroendocrine, immune, and inflammatory mechanisms in the gut, microbiome, and brain. Much research to date has elucidated the complex matrices of the microbiota-gut-immune-brain axis. In this narrative review, we aim to highlight the important role of the chronic stress response and its relationship to the microbiota-gut-immune-brain axis in the pathophysiology of psychiatric disorders, focusing primarily on human studies. To do so, we will address these complex mechanisms by discussing the multidirectional communications between the chronic stress response and the microbiota-gut-brain-axis, immune tolerance, and mental health (see **Figure 1**). Specific psychopathologies and their relationship with microbiome and immune dysregulation will be discussed, along with associated physical, mental, and nutritional triggers. Finally, we will briefly discuss potential prevention strategies and therapeutic targets.

## 2 The crossroads of chronic stress, immune activation, and the microbiome

### 2.1 Microbiota-gut-immune-brain axis

We coexist in an incredible symbiotic relationship with our commensal organisms, which contribute to the prevention or triggering of disease. The gut-brain axis (GBA) is a complex communication system that involves multiple interactions between gut functions and the emotional and cognitive centers of the brain. These interactions are mediated by various mechanisms, including neuro-immuno-endocrine mediators. The GBA includes the central nervous system (CNS), autonomic nervous system (ANS), enteric nervous system (ENS), and hypothalamic-pituitary-adrenal (HPA) axis (7). The HPA axis is considered the core stress axis that coordinates the adaptive responses of the organism to stressors of any kind. The gut microbiota plays an important role in the GBA, interacting not only locally with intestinal cells and ENS, but also directly with the CNS through neuroendocrine and metabolic pathways. Dysbiosis, an imbalance in the gut microbiota and therefore the gut microbiome, has been linked to various mental disorders, including anxiety, depression, and autism (8, 9). Dysbiosis also occurs in functional gastrointestinal disorders (FGID) that are strongly associated with mood disorders and have been linked to a disruption of the GBA. The ANS is a neural relay composed of a complex network with neurons located in both the CNS and peripheral nervous system. These are responsible for body functions that occur without conscious effort such as digestion, heart rhythm, and breathing. Combined with activity from the ENS and modulated by the CNS, the ANS promotes physiological homeostasis (7). To facilitate homeostasis, the ANS interfaces with endocrine, motor, autonomic, and behavioral areas, all of which comprise the larger network of the bidirectional GBA (7). The ANS works in conjunction with the other systems that comprise the GBA to enact CNS-driven changes to the gut (7). Communication between the gut and the CNS is mediated by the ANS. Because the vagus nerve (the tenth cranial nerve) directly innervates the gut, ENS, and ANS, it provides the most direct neurological response. This rapid neurological response may take the form of pain and stress responses (7). It follows then that ANS activity can induce ENS responses. Potential triggers to the ENS are often responsible for changes in gut motility, leading to differing delivery and uptake of microbiota-accessible carbohydrates (MACs) needed for the proliferation and maintenance of diverse gut microbiome composition. As part of the ANS circuit directly interfacing with the gut, the vagus nerve also serves as the fastest information exchange route between the gut and the CNS (7). The gut is enmeshed in hepatic and celiac branches of the vagus nerve (7). Density of these branches decreases caudally from the proximal duodenum, ileum, and the ileocecal junction; though, they continue to extend to the colon (7). The vagus nerve forms intraganglionic laminar endings, intramuscular arrays, terminal axonal endings in the mucosa, and neuropods, as a subset of enteroendocrine cells that comprise synapses with neurons of the vagus nerve (7).

The potential interplay between signaling molecules in the body and the microbial communities of the gut microbiome is another key feature of the bidirectional relationship between microbiota and behavior. The gut microbiota play a significant role in regulating the GBA and local and systemic immunity. The microbiome metabolites from the gut, particularly short-chain fatty acids (SCFA), have immunomodulatory properties and can interact with nerve cells by stimulating the ANS and the sympathetic nervous system (SNS) via G-protein-coupled receptors (10). SCFAs can also regulate the release of gut peptides from enteroendocrine cells, affecting gut-brain hormonal communication (10). The gut microbiota are capable of producing a variety of other neuroactive and immunomodulatory compounds, including dopamine, histamine, and acetylcholine (10, 11). Moreover, the gut microbiome is an important regulator of bile acid pool size and composition, which, in turn, affects blood-brain barrier (BBB) integrity and HPA function. The microbiota that constitutes the gut microbiome interact with bile salts in the gut to achieve this modulation of bile acid (7). Recently, certain taxa were shown to express bile salt hydrolase (BSH), which can deconjugate taurine and glycine from bile acids (7). The presence of microbiota expressing BSH in the gut microbiome has been linked to an increase in diversity of bile acids in the host gut (7). Additionally, deconjugated bile salts are shown to be less efficiently reabsorbed by the small intestine (7). This in turn has implications on host metabolism and weight gain (7). The gut microbiota may also contribute to the regulation of brain function by influencing tryptophan metabolism. Once absorbed from the gut, tryptophan can cross the BBB and participate in serotonin synthesis; however, the availability of tryptophan is heavily influenced by the gut microbiota (10, 11). Resident gut microbiota can utilize tryptophan for growth or, in some cases, production of indole or serotonin (10, 11). The gut microbiota could influence serotonergic neurotransmission by limiting the availability of tryptophan for serotonin production in the CNS (7, 11).

The gut microbiota also influences the development of the HPA axis and the stress response, which will be covered in detail subsequently. In short, activation of the HPA axis leads to the release of corticosterone releasing factor (CRF) from the hypothalamus, which stimulates the release of adrenocorticotrophic hormone (ACTH) from the anterior pituitary, inducing the synthesis and release of glucocorticoids from the adrenal cortex (12). Germ-free mouse models have shown that the microbiota play a key role in the development of the HPA axis and its stress response (12). Germ-free animals exhibit exaggerated HPA axis activity with elevated ACTH and corticosterone in response to stress, which is normalized (‘rescued’) after fecal microbiota transplant from control mice (12). The gut microbiota and stress response are also linked in humans, with probiotic supplements shown to improve stress and emotional responses (12).

### 2.2 The chronic stress response and mental health

Chronic stress exposure has severe lasting biological consequences (13). The field of psychoneuroimmunology, which elucidates the relationship between immune function, brain health, and psychosocial factors (14) has highlighted the impact of chronic stress exposure throughout the lifespan, from early life and prenatal stress to adulthood (15, 16). Threats come in many forms, including external (noise, physical abuse) and internal (illness, sleep deprivation, emotions) and are processed by the brain exteroceptively (from outside) and interoceptively (from within) to trigger the stress response (17). The acute response to real or perceived stress is an adaptive mechanism designed to protect the host and maintain homeostasis through neural, endocrine, and immune processes, primarily via the SNS and hypothalamic-pituitary-adrenal (HPA) axis (18). The (CNS), ANS (sympathetic and parasympathetic divisions) and HPA-axis work in concert to activate and deactivate the stress response as needed. However, chronic HPA-axis activation with associated cortisol dysregulation is maladaptive and associated with a myriad of negative health outcomes, including increased susceptibility to infection, metabolic syndrome obesity, cancer, cardiovascular disease, mental health disorders (18), and neurological disorders such as Alzheimer’s disease (19). In fact, the profound impact of psychological stress on inflammatory processes is an often-overlooked management strategy in numerous disease states (20).

In brief, the HPA-axis, SNS, and CNS are activated in response to stress with an ultimate release of catecholamines (i.e., epinephrine and norepinephrine), glucocorticoids (i.e., cortisol) and subsequent negative feedback to the CNS to prevent sustained activation and prolonged glucocorticoid exposure (19). Chronic exposure to stress alters neurotransmitter and hormone levels and increases immune activation centrally and peripherally, as evidenced by elevated proinflammatory cytokines (e.g., IL-6), increased monocytes and neutrophils, and activated microglia (21). Microglia, the resident immune cells in the brain, upregulate several immune markers (e.g., Iba1, CD11b, CD86, TLR4, CD14, and CD68), proinflammatory cytokines (e.g., IL-1β, CCL2), and reactive oxygen species (ROS), leading to phagocytosis of neuronal elements in response to stress (21). Furthermore, microglia highly express glucocorticoid receptors, pointing to their role in the stress response (22), and produce toll-like receptor 4 (TLR-4), for which the ligand is lipopolysaccharides (LPS), underscoring their involvement in immune and inflammatory processes as well as intestinal hyperpermeability (23). Microglia are key regulators of stress and neuroinflammation (22). With chronic activation, microglia promote sustained neuroinflammation, synaptic dysfunction, and altered brain network connectivity seen in various neuropsychiatric disorders (24, 25). Additionally, early life stressors may prime microglia, such that responses to stress are potentiated with accompanying neuroinflammation and susceptibility to mental illness (16). Early life stress during developmental periods, whether pre- or post-natal, is a predominant risk factor for an altered stress response and negative physical and neuropsychiatric outcomes, accounting for an estimated 45% of mental illness in children and up to 30% in adults (26).

**Figure 2:**
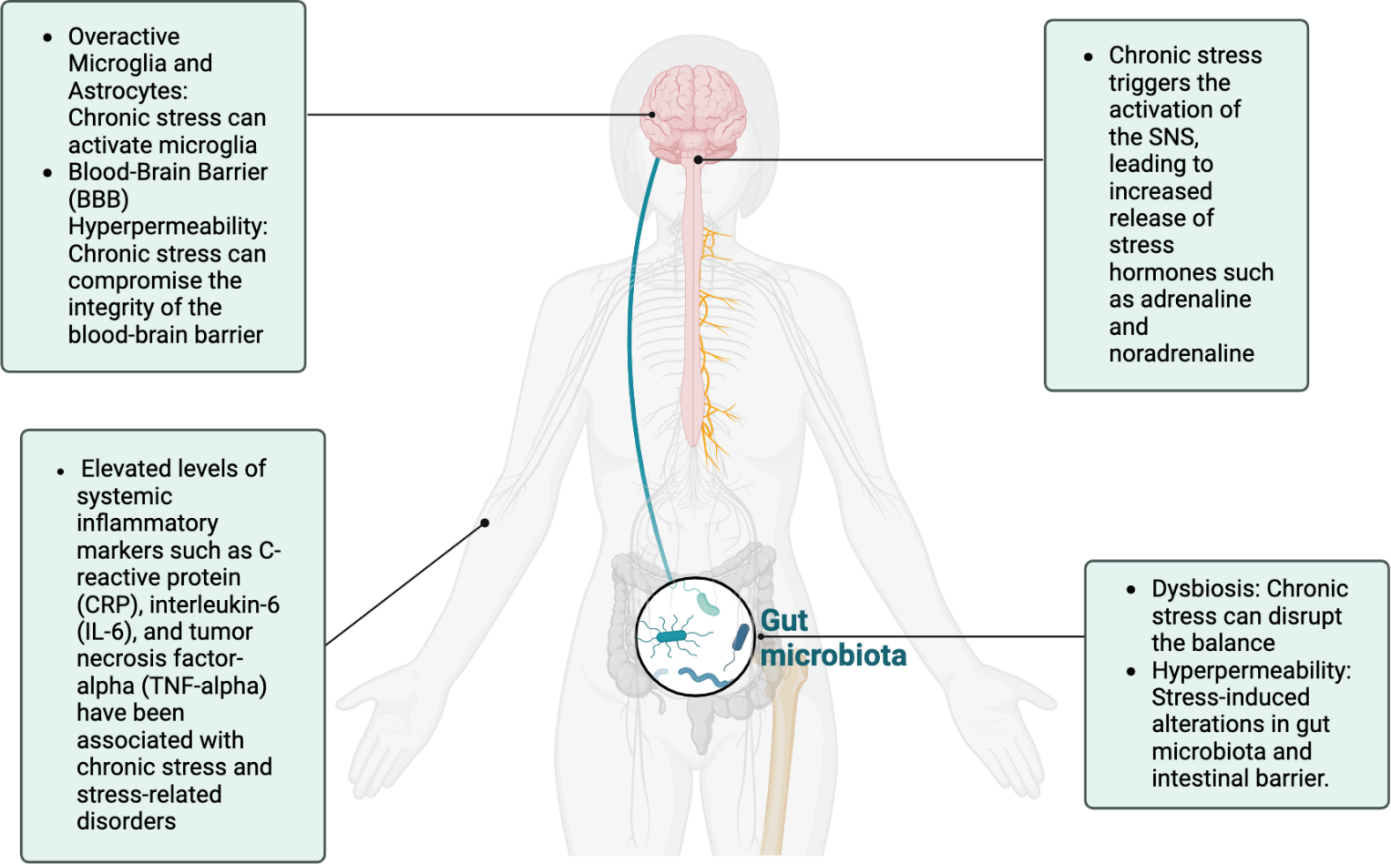
The chronic stress response, characterized by sympathetic overdrive and HPA-axis dysregulation, can have profound effects on inflammation across multiple systems, including the gut, systemic circulation, and the brain. These interconnected pathways underscore the bidirectional relationship between stress and inflammation, which plays a pivotal role in the pathophysiology of stress-related disorders.

Owing at least in part to altered immune responses and neuroinflammation, chronic stress exposure is one of the strongest risk factors for developing various psychiatric disorders such as burnout, depression, posttraumatic stress disorder, bipolar disorder, schizophrenia, anxiety disorders, substance abuse disorders, and addiction (5, 13, 21). Mounting evidence suggests that chronic stress and trauma can result in epigenetic changes that contribute to the development of psychopathology (13). Likewise, mental health has a profound effect on inflammatory processes throughout the body including the brain, indicating the importance of including mental health in the management of physical disorders and vice versa (20). These systemic and neuroinflammatory processes also contribute to dysbiosis via the microbiota-gut-immune-brain axis and are often associated with elevated IL-1β, IL-6, LPS, and decreased brain-derived neurotrophic factor (BDNF), a neurotrophin required for neuronal development and survival as well as synaptic plasticity and cognition (27). Dysbiosis also decreases concentrations of tight junction proteins (e.g., occludins, zonulin) in the intestine and BBB (23). As such, the intestine and BBB become hyperpermeable with compromised integrity–the “leaky gut, leaky brain” that can affect physical, cognitive, and mental health (whole-person well-being) (see **Figure 2**).

### 2.3 The chronic stress response and the microbiome

Microbiome research has emerged as a promising new frontier in disease and health. We are continuing to learn how chronic stress not only “gets under the skin,” but also “into the belly” and can further emerge *from* the belly (28). Evidence supports the critical role of gut microbes and their metabolites in central neurochemistry, mood, and behavior, particularly stress-related processes (6, 29), including but not limited to neurotransmitters, neuropeptides, SCFAs, bile acids, endocrine hormones, and immunomodulators (3). Notably, the ecosystem of the ENS shares many structural and chemical components, such as neurotransmitters, neurons, and glia. As previously mentioned, chronic stress leads to HPA-axis activation and ultimately, to dysbiosis, and the inflammation resulting from dysbiosis also leads to HPA-axis activation (27). *Via* the ENS by way of the microbiota-gut-immune-brain axis, psychological stress leads to chronically elevated glucocorticoid and results in monocyte- and TNF-mediated inflammation by inducing inflammatory enteric glia to promote monocyte recruitment via CSF-1, and consequential transcriptional immaturity in enteric neurons, acetylcholine deficiency, and TGF-b2-induced gut dysmotility (30).

The gut microbiome is a key player in both the top-down and bottom-up processes involving the relationship between neurobiology and the intestinal ecosystem, and clinically reflected in comorbidities of gastrointestinal (GI) symptoms with anxiety, depression, spectrum disorders, and neurodegenerative disorders (27). Stress therefore shifts the microbiome ecosystem into a state of dysbiosis that leads to intestinal and BBB hyperpermeability, endotoxemia, and neuroinflammation (31). This bidirectional dysbiosis-inflammatory-brain/mood connection can be observed in many disorders and is exemplified in inflammatory bowel disease (e.g., Crohn’s disease, ulcerative colitis), which are not only accompanied by psychological symptoms, but also exacerbated by psychological stress (30); this produces a vicious cycle of physical and mental symptoms seen in many disease states. Furthermore, as early life stressors may prime microglia to increase susceptibility to psychopathology, as discussed earlier, early life trauma may also prime the gut microbiome toward a state of dysbiosis and facilitate inflammatory cascades, thereby increasing the risk of post-traumatic stress disorder (32).

In addition to the immune and inflammatory contributions of the gut microbiome and stress, the gut microbiota also produce neurotransmitters including as gamma-aminobutyric acid (GABA), norepinephrine, epinephrine, dopamine, acetylcholine, melatonin, and histamine, which likely exert their effects via the ENS and vagal afferents (33–37). An estimated90% of serotonin alone is produced in the gut, primarily from enterochromaffin cells (36). Many psychopathologies and neurological disorders are associated with disruptions in neurotransmitter levels, which can also act as hormones affecting numerous receptor sites (38). While still under exploration, it has been shown that bacterial species from the gut microbiota dramatically alter chemicals that mediate neurotransmitters and neural transmission (38). Serotonin, which affects both mood and GI function, is the most studied neurotransmitter in the field of psychiatry (39) and is directly related to microbiota composition (38). Serotonin dysregulation is associated with anxiety and depression, and studies have shown that supplementation with probiotics from the genus *Bifidobacterium* and *Lactobacillus* may improve the symptoms of depression (39). Understanding the mechanisms behind microbiome perturbations that lead to dysregulation of neurotransmitters is a potential target for prevention and management of several psychopathologies, some of which will be discussed in following sections.

### 2.4 Immune dysregulation leads to autoimmunity

Immune dysregulation is a complex phenomenon that is key to the pathogenesis of autoimmunity. The immune system is designed to defend against pathogens, largely through antigen recognition, while at the same time maintaining tolerance to self by limiting self-reactive lymphocytes (reactive to self-antigens). This is maintained in two parts. First, the complex gene rearrangements during lymphocyte development, which lead to a diverse pool of antigen receptors, allow for robust immune responses to even yet unseen pathogens (40). Second, these lymphocytes are tested for self-tolerance and those that fail are eliminated or at least held in check either during development in the primary lymphoid tissue (central tolerance) or after leaving the primary lymphoid tissue (peripheral tolerance) under normal circumstances (40).

Autoimmunity occurs due to escape from the normal self-tolerance screening and/or elimination process (central and peripheral tolerance). This results in an aberrant immune reaction to self, which is termed autoimmunity. It is well known that genetics are an important predisposing factor for autoimmunity; this affects the ability of the immune system to perform the processes of central and/or peripheral tolerance (40). The environment is also a contributor to the ability of the immune system to maintain tolerance including infection, stress, and environmental exposure to certain chemicals. Additionally, this loss of tolerance can propagate through epitope spreading, in which the immune response expands to include additional self-antigens, and molecular mimicry, when foreign antigens share structural similarities with self-antigens resulting in confusion between foreign and self-antigens (40).

It is impossible to discuss immune tolerance without highlighting regulatory T cells (Tregs), a subset of T lymphocytes that promote tolerance and resolution of immune responses (return to homeostasis). Tregs can extrinsically mediate autoimmunity, as they are able to suppress self-reactive lymphocytes. This is regulatory tolerance, a type of self-tolerance. Further, Tregs can stimulate peripheral tolerance when antigens are delivered orally (oral tolerance), providing a potential mechanism for therapies against issues as diverse as food allergies and autoimmunity (40). A balance between the classically activated M1 (Th1 responses) and alternatively activated M2 (Th2 responses) macrophages is also important in the role of immune tolerance. M2 macrophages produce IL-10 and TGF-beta to promote wound healing (40) and can also play a role in immunosuppression. A complex system of checks-and-balances is required to have a robust immune response without autoimmunity. Therefore, for autoimmunity to be established, multiple mechanisms must fail; this is further exacerbated by an inflammation positive feedback loop (40).

## 3 Stress, psychopathology, and the microbiome

Dysbiosis and psychopathology appear to be related in many ways. The literature suggests a dual relationship, functioning in a manner through which changes in gut microbiota affect behavior, while conversely, changes in behavior result in alterations in gut microbiome composition (41, 42). Of note, medications used to treat mental disorders are often associated with GI side effects, suggesting further effects on the gut microbiome (43). Likewise, evidence suggests that certain probiotic strains, fecal microbiota transplantation (FMT), prebiotics, postbiotics (i.e., SCFA), and dietary modifications can alleviate some symptoms associated with mental illness (44), although evidence is limited and research ongoing. Many psychiatric disorders have been shown to have significant differences in microbiome composition (see **Table 1**), including depression, bipolar disorder, schizophrenia, autism spectrum disorder, attention deficit hyperactivity disorder (ADHD), and pediatric autoimmune psychiatric disorder associated with streptococcal infections (PANDAS) (44, 45). It is important to note, however, that much of the available literature involves animal models and considerable differences in assessment methods. At present, mechanistic contributions, at least in animals, seem to be clearer than appropriate intervention strategies in human studies.

**Table 1:** Examples of microbial alterations in pyschopathology.

Depression and Anxiety	Lower levels of *Faecalibacterium*, *Dialister*, *Coprococcus* spp.. *Flavonifractor* (formerly *Eubacterium*) was increased in participants with depression
Obsessive Compulsive Disorder	Lower diversity and abundance of butyrate-producing genera, specifically *Pseudomonas*, Caulobacteraceae (family), *Streptococcus*, *Novosphingobium*, and *Enhydrobacter*
Bipolar Disorder	Lower abundance of the phylum Bacillota (formerly Firmicutes) specifically *Faecalibacterium*, the family Lachnospiraceae, and the genuses *Akkermansia* and *Sutterella*
Schizophrenia	Noted presence of the phylum Pseudomonadota and increased lactic acid bacteria
Alcohol Use Disorder	Lower abundance of *Akkermansia muciniphilia*, *Faecalibacterium prausnitzii*, *Bacterioides*, and at the phylum level more Pseudomonadota (formerly Proteobacteria), and more members of the Enterobacteriaceae family

### 3.1 Depression & anxiety

The incidence of depression and anxiety has significantly increased over the past several decades (46). Depression is the leading cause of medical disability worldwide (47) and major depressive disorder (MDD), the more severe form, is the second leading cause of disability in the United States; both are associated with pathological shifts in gut microbiota (47). The relationship between the gut microbiome and depression and anxiety was established more than a decade ago but continues to be refined (48).

Recent work suggests that the microbiota-gut-brain axis functions in a bidirectional manner in the regulation of depressive-like behaviors (7, 11). Data from mouse models demonstrate that changes in behavior caused by stress, knockout of caspase-1, or pharmacological treatments result in changes in the gut microbiome (41). In germ-free mice, the absence of gut microbiota results in decreased immobility time in the forced swimming test compared to conventionally-raised, healthy, control mice (41). From clinical sampling, gut microbiome composition significantly differed between MDD patients and healthy controls (7, 42). FMT from MDD patients to germ-free mice resulted in depression-like behaviors compared with FMT from healthy controls (12, 41). Generally speaking, MDD is associated with reduced numbers of *Bifidobacterium* and *Lactobacillus* versus healthy controls (12).

Ghrelin is a gut hormone that regulates energy homeostasis, eating and sleeping behavior, cognition, reward mechanisms, and mood (7, 49). Ghrelin interacts with acylated ghrelin receptors expressed by the hypothalamus, dentate gyrus, and other regions of the brain. This hormone has been shown to regulate mood and has close links to depression (49). Both acute and chronic stress results in elevated ghrelin, whereas prolonged stress also leads to chronic increased dysregulation of the HPA axis, serotonin signaling, and increased depressive behaviors–perhaps due to prolonged overexpression of ghrelin. Ghrelin has been associated with MDD, and elevated ghrelin has been found to act as a measure of treatment response (7, 42). Ghrelin is primarily produced in the gut, and germ-free mice have lower circulating ghrelin (7, 41, 49). Prebiotic treatment, which increases SCFA production, has been found to alter ghrelin, the production of which is regulated by SCFA signaling (7). Gastric infusion of the SCFA acetate increases plasma ghrelin, indicating potential bidirectional communication from the gut to the brain in the control of ghrelin secretion from cholinergic cells.

Due to its role in mediating mood and its connection to the gut microbiota, ghrelin could serve as an optimal biomarker to identify treatment response to prebiotic and probiotic treatment in the MDD population (7, 42).

Patients with irritable bowel syndrome (IBS) and inflammatory bowel disorders (IBD) are at higher risk of depression (50). The relationship between dysbiosis and MDD has been hypothesized to involve the microbiota-inflammasome-brain connection, whereby dysbiosis caused by stress and GI conditions exacerbates MDD via upregulation of pro-inflammatory pathways including the caspase-1-dependent, pro-inflammatory NLRP3 inflammasome present in several immune cell types (e.g., microglia, monocytes, granulocytes, T cells, B cells) (51). Furthermore, C-reactive protein (CRP), which tends to be chronically elevated in depression and anxiety, has been shown to interact with the gut microbiome and affect the risks of anxiety and depression (46). Messaoudi et al.found that taking a probiotic formulation of *Lactobacillus helveticus* R0052 and *Bifidobacterium longum* R0175 for 30 days decreased anxiety and depression scores while concomitantly lowering cortisol (52). A more recent study by Valles-Colomer et al.surveyed a large microbiome cohort from the Flemish Gut Flora Project (n=1,054) to examine the relationship between microbiome alterations and quality of life in depression (53). They found that butyrate-producing bacteria (e.g., *Faecalibacterium* and *Coprococcus*) were associated with higher quality of life measures: *Flavonifractor* was increased in depression; *Dialister*, *Coprococcus* spp. were reduced in depression (53). Further,the dopamine metabolite 3,4-dihydroxyphenylacetic acid was positively correlated with quality of life, suggesting the potential role of microbial neurotransmitter production in depression (53).

Furthermore, changes in serotonergic signaling in germ-free mice may contribute to the altered anxiety-related phenotype observed (12, 41). Studies in germ-free animals have shown that microbial colonization of the gut is central to the development and maturation of both ENS and CNS (41). The absence of the gut microbiome is associated with altered expression and turnover of neurotransmitters in both nervous systems and neuromuscular abnormalities (12, 41). These anomalies are restored after microbial colonization in a bacterial species-specific manner; in other words, the germ-free animals are rescued by gut microbiome colonization. This link reinforces the bidirectional mechanism of the GBA and the role that the microbiome plays in influencing the development of behavior in mice, with germ-free mice exhibiting an anxiolytic phenotype in the elevated plus maze (EPM), an ethologically and pharmacologically validated tool for the assessment of rodent anxiety-like behavior (12, 41, 54). The molecular data provide initial insights into the neurobiological pathways underlying this behavioral phenotype. In germ-free mice the subunit of N-methyl-D-aspartate (NMDA) receptors, NR2B, was severely downregulated. This downregulation was particularly acute in the central amygdala and is thought to contribute to the anxiolytic-like phenotype that was observed in the EPM (41). NMDA receptors are heteromeric complexes and are made up of both NR1 and NR2 subunits. The NR2B subtype is the critical receptor in amygdala synaptic plasticity and development and in learning and memory. Additionally, NMDA receptor antagonists are known to block anxiety-like behavior in both mice and rats (12, 41, 54). Antagonists specific to NR2B block the acquisition of amygdala-dependent fear-learning, further illustrating the role that this NMDA receptor subtype plays in the expression of anxiety, fear, and CNS plasticity. Up-regulation of BDNF mRNA in the dentate region of the hippocampus in the germ-free mice is consistent with literature identifying a role for this molecule in anxiety-like behaviors (41). Recent work has demonstrated that impaired BDNF signaling in the dentate gyrus of adult mice results in a marked increase in anxiety-like behavior (41). Hence, it is reasonable to suggest that increased BDNF may be related to the observed reduction in anxiety-like behaviors in germ-free mice.

### 3.2 Obsessive compulsive disorder

Obsessive compulsive disorder (OCD) is a chronic debilitating mental illness with an unclear etiology involving immune, neurotransmitter, endocrine, and microbiome dysregulations (45). Immune status affects the evolution of OCD and is marked by an alteration in the type of immune response, especially Th1 versus Th2 (55). In concert with HPA-axis dysregulation, cytokines such as IL-6 and TNF-α tend to be increased in OCD (55). However, a recent study suggests that CRP may be more clinically relevant than IL-6 and TNF-α. Turna et al. compared the microbiomes and inflammatory markers of 21 non-medicated OCD patients to 22 age- and sex-matched controls and found elevated CRP compared to controls, but not IL-6 or TNF-α (45). Further, CRP was associated with severity of symptomatology and the OCD group presented lower diversity and abundance of butyrate-producing genera in their gut microbiomes (45). Larger studies are needed for clarification.

A more recent study by Kang et al., compared the microbiome of OCD patients to health controls using circulating bacterial extracellular vesicles in serum and found that at the genus level *Pseudomonas, Caulobacteraceae(f), Streptococcus, Novosphingobium*, and *Enhydrobacter* were significantly reduced in the OCD group, and microbial composition of the genera *Corynebacterium* and *Pelomas* were significantly different in the early-onset versus late-onset types (56).

### 3.3 Bipolar disorder

Bipolar disorder is a serious mental disorder that has been associated with systemic immune alterations and chronic inflammation, mediated by the gut microbiota (57). Some evidence suggests that microbiome alterations are associated with bipolar disorder, particularly a decrease in Bacillota, formerly Firmicutes, especially *Faecalibacterium* (3). Surprisingly, pharmacological treatment of bipolar disorder may further exacerbate dysbiosis. In a study involving atypical antipsychotics in females with bipolar disorder, the treatment group demonstrated decreases in the family Lachnospiraceae and in the genuses *Akkermansia* and *Sutterella* compared to treatment-naïve controls (58). What’s more, patients with bipolar mania are almost twice as likely to have been recently treated with systemic antibiotics, and studies examining probiotic use suggest that it could reduce the rate of re-hospitalization following a manic episode (3). More research is warranted and needed.

### 3.4 Schizophrenia

Schizophrenia is a lifelong, serious mental disorder and, like many other pathologies, is associated with immune dysfunction, chronic inflammation, and dysbiosis (57). In the limited studies of patients with schizophrenia, the gut microbiome has shown increases in the phylum Pseudomonadota, formerly Proteobacteria, (predominantly the genus *Succinivibrio*), increased abundance of lactic acid bacteria and *Lactobacillus* phage in the oral microbiome, and a relationship between dysbiosis and first episode psychosis severity (44). Further alterations have been observed but a clear pattern has not yet emerged. While speculative, dysbiosis may be related to psychosis as a result of microglial activation through SCFA production alteration (44).

### 3.5 Alcohol use disorder

Alcohol use disorder (AUD) is one of the most significant preventable contributors to morbidity and mortality (59, 60). Alcohol directly alters the permeability of the intestinal barrier, causes dysbiosis, and is associated with peripheral and central inflammation (60). A systematic review of 17 studies of AUD and the gut microbiome by Litwinowicz et al. found that individuals with AUD demonstrated lower abundance of *Akkermansia muciniphilia* and *Faecalibacterium prausnitzii*, less of the genus Bacterioides, at the phylum level more Pseudomonadota (formerly Proteobacteria), and more members of the Enterobacteriaceae family (61). A recent study by Litwinowicz & Gamian compared the microbiomes of participants with AUD, alcoholic liver disease (ALD), and healthy controls (59).

They found a significantly lower abundance of butyrate-producing families (especially the species *Ruminococcaceae*, *Lachnospiraceae*, and *Oscillospiraceae*) in AUD compared to controls, which was more severe in ALD, and, at the phylum level, an increase in endotoxin-producing Pseudomonadota (formerly Proteobacteria*)* in AUD, again worse so in ALD compared to controls. Fungal microbiome studies have suggested significantly increased abundance of the genera *Candida*, *Debaryomyces, Pichia, Kluyveromyces*, and *Issatchenkia* and of the species *Candida albicans* and *Candida zeylanoides* compared to controls, some of which decreased with alcohol abstinence (62).

Therefore, there is the potential for gut microbiome targeted therapeutics to support management of AUD and ALD with substantial additional research necessary.

## 4 Immune dysregulation and the microbiome

### 4.1 Autoimmunity and the microbiome

Immune dysregulation can result from inappropriate immune reaction to commensal microbiota, the residents of human microbiomes. When this reaction causes disease, it is technically termed xenoimmunity, as the microbiota are not built by the host but rather acquired from foreign sources (e.g. maternal microbiome, dietary intake, the environment, etc.) (40). However, the presentation of such disease is virtually identical to autoimmunity. Further, many are beginning to consider the various microbiomes as vital components of their host, meaning they cannot be separated from the host. Thus, immune responses to commensal microbiota that result in disease are typically included in the autoimmunity category, and we will continue to refer to these as autoimmunity here. The classic example of autoimmune disease stimulated by a microbiome is Crohn’s disease, a type of IBD in which T lymphocytes react to commensals in the gut microbiome (40). Crohn’s disease is characterized by waves of severe inflammation, diarrhea, pain, and fatigue and often results in weight loss, malnutrition, and granulomatous lesions in the intestinal mucosa and submucosa. There is a strong genetic component to Crohn’s disease including NOD2, a gene that regulates lymphocytes and antigen recognition in the intestine through autophagy (recycling of cellular components or whole cells) (63).

Further, the microbiota plays an important role in immune education, the process by which an immune system learns to recognize appropriate targets, mount a robust immune response, and return to homeostasis accordingly (40). Without proper immune education during the early life window of opportunity, aberrant immune responses are more likely including allergy and autoimmunity (64–66). Therefore, microbiota may be both a trigger for self-reactive lymphocytes and for aberrant immune responses required to support autoimmunity.

All of this contributes to function or dysfunction of the microbiota-gut-immune-brain axis, which is exaggerated by an inflammation positive feedback loop. Multidirectional links have been made between the gut microbiota, the HPA axis, and mental health issues; however, this work has been mostly in animal models, which have translated very poorly to humans, especially in this field, to-date (66, 67). Therefore, caution is warranted when interpreting the literature until more translational research is conducted showing strong relationships in humans. At that point, animal models will be very useful for mechanistic studies.

### 4.2 Physical/mental triggers of autoimmune reactions and dysbiosis overlap

Several physical and psychological triggers have been associated with the dynamic interplay between immune dysregulation, autoimmunity, inflammation, and dysbiosis – the effects of which are widespread and far-reaching. In addition to psychological stressors and trauma discussed previously, physical triggers have also been associated with this detrimental cycle. Examples of environmental triggers include diet, alcohol, medication, infections, pollution, physical activity, and calorie intake (68). In genetically predisposed individuals, environmental triggers can result in loss of self-tolerance, and dysbiosis may be one important pathway by which the immune system erroneously favors proinflammatory pathways that instigate autoimmune states (69). Recently, novel research questions are exploring the role of microbiome-induced autoimmunity as a novel pathoetiological factor, primarily involving intestinal hyperpermeability, dysbiosis, toll-like receptor (TLR) ligands, and B cell dysfunction, as well as potential therapeutic implications (70). Altered microbial composition and this inflammatory-dysbiosis-autoimmune process has been identified in a number of autoimmune diseases, such as IBD, multiple sclerosis, lupus, rheumatoid arthritis, and type I diabetes (69, 71–74). This lends further credence to the common neuropsychiatric comorbidities in autoimmune and other inflammatory states (71). A predominance of Th1/Th17 lymphocytes, plasma cells, and antigen presenting cells (APCs) can instigate the process by presenting luminal microbiota-derived antigens and toxins to T and B lymphocytes, which then become inappropriately activated in autoimmune states (69). Probiotic and FMT studies have shown some promise but need further refinement to establish clear therapeutic recommendations. Nonetheless, treatment with probiotics, prebiotics, and other microbiome-altering therapies may facilitate eubiosis and symptom management in autoimmune diseases (74) by utilizing gut microbes to promote immunomodulatory responses that balance autoimmune-related inflammation (73).

### 4.3 Nutrition contributions to dysbiosis and inflammation

Certain dietary components have been identified as pro-inflammatory, contributing to an increase in inflammatory markers. These include saturated fats, trans fats, refined sugars, and excessive intake of omega-6 fatty acids, commonly found in ultra-processed and fast foods, over omega-3 fatty acids, especially the non-essential omega-6 fatty acids (68).

Saturated fats, commonly found in red meat and ultra-processed foods, have been associated with increased production of pro-inflammatory molecules. In contrast, monounsaturated fats found in olive oil, seeds, nuts, and legumes and omega-3 fatty acids present in fatty fish, seafood, seeds, nuts, and legumes exert anti-inflammatory effects (68). Refined carbohydrates, such as those found in sugary beverages and white bread, can contribute to inflammation while whole grains, rich in fiber and antioxidants, have been linked to lower inflammatory markers (44, 68). Anti-inflammatory foods, such as vegetables, fruits, whole grains, and omega-3 fatty acids, possess properties that help modulate the immune response and reduce inflammation (68). In fact, omega-3 fatty acids are necessary to turn off the inflammatory process via specialized pro-resolving mediators (SPMs) (75, 76). Antioxidants found in vegetables and fruit play a crucial role in mitigating inflammation by neutralizing free radicals; these compounds include vitamins C and E, beta-carotene, and various polyphenols.

Research by David et al. demonstrated that a short-term shift to a low-fiber, high-fat diet could rapidly induce significant changes in the composition of the gut microbiome. This shift was marked by a decrease in beneficial bacteria, such as *Bifidobacteria*, and an increase in potentially harmful bacteria, including members of the phylum Bacillota, formerly Firmicutes (75). These alterations are indicative of dysbiosis and inflammation. Another study by Esposito et al. demonstrated that a Mediterranean diet, rich in fruits, vegetables, whole grains, and omega-3 fatty acids, significantly reduced inflammatory markers in individuals with MDD (76). Conversely, diets high in refined sugars, saturated fats, and ultra-processed foods have been associated with increased inflammation. Additional studies from other researchers studying inflammatory diseases offer support for the important role of nutrition in dysbiosis and inflammation. Attur et al. and de Oliveira et al. studied the role of intestinal dysbiosis and nutrition in the context of inflammatory diseases such as rheumatoid arthritis (RA) (77, 78). These studies demonstrated the intricate connections between the gut microbiome, dietary patterns, and systemic inflammation, emphasizing the potential link between diet-induced microbial changes and the pathogenesis of RA. Gill et al. demonstrated how various diets impact the gut microbiota and lead to inflammation, which plays a crucial role in GIand inflammatory diseases (79).

Potrykus et al. demonstrated microbial contributions to chronic inflammation and proposed potential modifications of the gut microbiome as therapeutic interventions (80, 81). Their work suggested that targeted microbiome specific strategies to modulate dysbiosis-related inflammatory responses can be explored as a potential treatment strategy for inflammatory diseases and other disorders linked to the inflammation. Taken together, the evidence suggests that nutrition can contribute to dysbiosis and inflammation, which can in turn impact the GBA and the development of mental health disorders. Ouabbou et al. describe the microbiome as “a potential missing link” when studying the impact of inflammation in mental disorders (80). This suggests that the intricate relationship between inflammation and the gut microbiome plays a crucial role in mental health conditions like depression and anxiety (82).

Vitamin D, classically known for its role in bone health, has been established as an immunoregulatory hormone and is now being linked to the gut microbiome. Seasonal Affective Disorder (SAD), a subtype of depression with a seasonal pattern, is characterized by recurrent depressive episodes during specific seasons, linked to reduced sunlight exposure. Seasonal changes in sunlight impact vitamin D synthesis, and vitamin D deficiency has been associated with increased inflammation (83). Vitamin D deficiency is associated with dysbiosis (84), suggesting a role for vitamin D in microbiome dysbiosis. Further, as an immunoregulatory hormone, vitamin D impacts the immune response in general and likely specifically to commensal microbiota. There has been limited research on this specific topic, but there is strong mechanistic plausibility.

## 5 Prevention strategies and therapeutic targets

A major advantage of microbiota-targeted therapy lies in the dynamic nature of the microbiome that can be readily altered by several interventional strategies, such as diet, exercise, and stress management (3). Dietary interventions have long been known to affect inflammation and continue to serve as a main lifestyle intervention for the prevention and treatment of various diseases. Nutritional psychiatry is one field of research that recognizes the impact of nutrition on the brain, mood, behavior, and mental health and respective methods to improve mood and treat mental illness (85).

The Western diet, for example, is associated with increased depression risk while the Mediterranean diet reduces the risk (12). Dietary origins of mood changes and inflammation have ignited novel approaches to the treatment of depression, for example, with polyunsaturated fats (PUFA) (86). Serotonin production and release in the gut, for example, is largely the product of dietary choices such as complex carbohydrates and tryptophan containing foods (87). The anti-inflammatory effects mediated by microbial metabolites of dietary fiber and polyphenols confer multidirectional benefits to brain and mental health and hold the potential to serve as nonpharmacological approaches to mental illness to improve treatment outcomes (87). Population studies and clinical trials support positive effects of diet in mood disorders such as depression and anxiety, even in severe presentations (85). While specific micro- and macronutrients are important considerations, consuming a wide variety of nutritious foods has been demonstrated to provide the most beneficial effects on physical and mental health, rather than a focus on single nutrients that are not reflective of real-life eating habits (87).

The Mediterranean diet is one example of a dietary lifestyle that has demonstrated efficacy in decreasing the risk of depression in numerous studies, including randomized controlled trials (88–90), including in older adults (91). One of the more recent randomized controlled trials (HELFIMED) examined the Mediterranean diet supplemented with fish oil in 152 adults suffering from depression and found the intervention group had greater reduction in depression and improved mental health (88). They also found increased vegetable diversity, nuts, and legumes, along with increased omega-3 fatty acids, decreased omega-6 fatty acids were all correlated with improved mental health (88).

The Mediterranean-DASH diet Intervention for Neurodegenerative Delay (MIND), which emphasizes green leafy vegetables, berries, and low intake of red meat, is commonly recommended to prevent and slow cognitive decline, has also been inversely associated with odds of depression and psychological distress (92). A recent prospective cohort study in older adults found that high adherence to the MIND diet was associated with lower rates of depression over time (93).

Considering the influence of the microbiome on the brain, mood, and behavior, as well as altered eating habits and weight gain that often occur with stress-related mental disorders (e.g. MDD, PTSD), diet and nutrition are therapeutic strategies worth consideration, especially as adjunct therapy (86).

Taking into account the considerable role of the chronic stress response in dysbiosis and mental illness, stress management techniques are a vital component of whole-person well-being. Herein lies another way in which the microbiota-gut-immune-brain axis may be regulated, although this research area is still in its nascency. Cognitive behavioral therapy (CBT) is a well-established intervention for various psychological disorders and is a first-line treatment for anxiety-related disorders (e.g., generalized anxiety disorder, social anxiety disorder, phobias, OCD, PTSD) (94). An interesting recent study by Jacobs et al. examined the effect of CBT in 84 IBS patients on IBS symptoms and the microbiome (93). Prior to CBT, participants had increased fecal serotonin, increased the order Clostridiales and decreased *Bacterioides*, whereas post CBT participants demonstrated improved functional connectivity-related brain changes that correlated with *Bacteriodes* expansion (95). Mindfulness and meditation are mind-body techniques that are utilized for stress management and a broad range of disorders, and therefore also merit mention in this regard. Mindfulness is posited to facilitate a healthy gut microbiome and gut-barrier function by its ability to reduce inflammation and modulate the stress response (96). More specific microbiome-mind-body intervention studies are beginning to emerge. A recent study by Wang et al. examined the efficacy of mindfulness-based cognitive therapy (MBCT) in high trait anxiety individuals and its impact on gut microbiota in 21 young adults with high trait anxiety compared to 29 healthy controls (97). In the high trait anxiety group they found markedly decreased bacterial diversity with distinct clusters (significant overgrowth of *Streptococcus*, *Blautia*, and *Romboutsia;* decreased *Faecalibacterium, Coprococcus*,and *Lachnoclostridium*) compared to healthy controls. They also found that the MBCT intervention decreased anxiety and depression, improved mindfulness and resilience, and shifted microbial populations to more similarly diverse profiles as the healthy controls (97). The experience of stress is also affected by one’s daily environmental conditions and lifestyle habits, such as physical activity, screen time, and time outdoors. The literature suggests that intentional exposure to outdoor environments, in the form of outdoor walks and exercise, gardening, and nature viewing may reduce the experience of stress and improve well-being (98). This is a critical consideration for those who have restricted access to outdoor environments, such as those living in facilities for mental illness or cognitive decline.

Exercise confers innumerable health benefits, the mechanisms by which far exceed the scope of this paper. The physiological processes of stress and inflammation reduction are firmly established and continue to unravel additional connections, such as microbiota involvement. Research is beginning to support a mutual benefit of antioxidant overexpression and exercise on the microbiome (99). Exercise also serves to modulate several metabolic processes and neurotransmitters related to metabolic, psychological, and gut health (100). Exercise therefore occupies an important role in mitigating the stress response and health of the microbiota-gut-immune-brain axis.

## 6 Conclusion

This brief narrative review highlights several compelling research areas that support the complex matrix of the chronic stress response, immune dysregulation, mental illness, and the microbiota-gut-immune-brain axis. Many of these topics merit their own in-depth review, but the mechanistic insight of a systems biology approach to mental and autoimmune disorders may inform clinically relevant approaches to prevention and management strategies. Chronic stress is a key constituent in a multitude of negative physiological and psychological consequences, and although managing the stress response is far from a novel idea, the ways in which we intervene to treat mental health disorders and avoid physical consequences are sorely needed. Incorporating the role of the microbiome into this dynamic interplay is one avenue with which the clinical landscape can shift from reactive to proactive.

## Author contributions

AW: Conceptualization, Project administration, Writing – original draft, Writing – review & editing, Supervision. YN: Conceptualization, Project administration, Writing – original draft, Writing – review & editing. AB: Writing – original draft, Writing – review & editing. LAF: Conceptualization, Project administration, Writing – original draft, Writing – review & editing.
